# Egg production performance and quality in laying hens fed a diet rich in bakery by-product

**DOI:** 10.1016/j.psj.2026.107266

**Published:** 2026-06-10

**Authors:** Niko Gioacchino Zeni, Vera Perricone, Cristina Tognoli, Silvia Cerolini, Annalaura Lopez, Achille Schiavone, Stefano Paolo Marelli, Andrea Giorgino, Federico Fiamberti, Paolo Rosso, Luisa Zaniboni

**Affiliations:** a Department of Veterinary Medicine and Animal Sciences (DIVAS), University of Milan, Via dell’Università 6, Lodi, LO 26900, Italy; b Department of Veterinary Sciences (DSV), University of Turin, Largo P. Braccini 2, 10095, Grugliasco, TO, Italy; c Regardia, Via Sperina Alta 18, 12030, Marene, CN, Italy; d PBA Mangimi S.r.l., Via Gerole 1, 26861, Fombio, LO, Italy

**Keywords:** Laying hen, Bakery by-product, Former foodstuff, Egg production, Egg quality

## Abstract

The poultry sector faces increasing sustainability challenges related to feed-food competition, volatility in the cereal markets and the environmental impact of poultry production. In this context, the use of products from the bakery and pasta industry (PBPI) represents a promising strategy within a circular economy framework. The present study aimed to assess the effects of the dietary inclusion of commercial PBPI on productive performance and egg quality in laying hens. The commercial product was composed of bakery by-products, pasta, chocolate residues, and industrial confectionery by-products.

From 21 to 35 weeks of age, a total of 180 Lohmann Brown® laying hens received one of three dietary treatments: a control diet (CD), or a diet in which 20 or 40% of the CD was replaced with PBPI (PBPI20 and PBPI40, respectively). All three diets were isoenergetic and isonitrogenous. The study was designed to assess feed intake, body weight, egg production parameters and physical traits, and the chemical composition and fatty acid profile of eggs.

The dietary inclusion of PBPI did not affect egg number or oviposition rate. However, egg mass production was significantly reduced in hens fed PBPI40 compared with CD and FFs20. ADFI increased in both groups fed PBPI, resulting in higher FCRs, although overall production efficiency remained satisfactory. Egg quality was largely unaffected by PBPI20, whereas PBPI40 reduced eggshell weight and shell and yolk color intensity. From a chemical perspective, the eggs from hens fed PBPI showed a reduced proportion of saturated fatty acids and an increased content of C18:3n-3, although the overall n-6/n-3 ratio was not significantly modified.

In conclusion, the inclusion of PBPI in laying hen diets provides a viable and sustainable feeding strategy. A 20% inclusion rate had no impact on either productive performance or egg quality and resulted in a partial improvement in the nutritional profile of eggs. The present results support the valorization of PBPI as feed ingredients and demonstrate their capacity to enhance the sustainability of poultry production systems.

## Introduction

Cereals constitute the primary feed ingredient for the most prevalent intensive livestock species, including poultry species, pigs, and cattle, and approx. one-third of the global cropland dedicated to cereals is used for animal feed production (Garnett, 2009; Muscat et al., 2020). The poultry sector is the most significant in terms of the demand for cereals, accounting for 44% of the cropland used for livestock feeding (Sandrucci et al., 2018). In addition, the utilization of edible crops for both human consumption and livestock feeding generates competition for limited resources, a dynamic commonly described as feed–food competition (Breewood and Garnett, 2020).

Alternative feed ingredients and strategies that can support animal nutrition and reduce dependence on conventional raw materials, competition for natural resources, and food waste accumulation are urgently required to improve feed efficiency and sustainability in the livestock sector (Pinotti et al., 2021; Govoni et al., 2021). In poultry farming, grain accounts for 50% of the feed ration, thus its substitution with upcycled ingredients could significantly contribute toward improving the sustainability of the sector and reducing feed–food competition (Mottet et al., 2017). Moreover, the dependence on cereals exposes the poultry industry to fluctuations in market prices, ultimately increasing the cost of poultry products for consumers (Mottet and Tempio, 2017). According to the OECD/FAO (2025), the demand for poultry products is projected to grow by 21% by 2034, implicating a parallel increase in the pressure on feed resources.

One promising strategy to alleviate the pressure on feed resources for intensively produced monogastric animals, including poultry, involves the utilization of raw materials produced as part of the human food chain. These food materials have been identified as former foodstuffs (Pinotti et al., 2019), by-products (Yang et al., 2021), co-products (Van Zanten et al., 2014), food leftovers (Pinotti et al., 2021) or bakery by-products (Mahmoud, 2017). The use of these materials has the potential to reduce the consumption of natural resources for animal feed production and, consequently, its carbon footprint (Pinotti et al., 2023). According to the European Union Regulation, they are defined as products from the bakery and pasta industry (PBPI) in the catalogue of raw materials. The PBPI include products obtained during and from the production of bread, biscuits, wafer or pasta (European Commission, 2022). It is particularly relevant to consider that, in Europe, a production of ∼5 million tons annually of PBPI is used in animal nutrition, representing significant potential for the livestock sector (EFFPA, 2022).

In addition, the use of PBPI implements the concept of circular economy, a key priority for the European Union and coherent with the sustainable development goals recently adopted by the United Nations (2023). In this way, food waste is recycled and converted into an economic resource with a consequent reduction in the environmental impact of livestock production (Luciano et al., 2020).

The nutritional characteristics of PBPI may vary considerably according to the origin and composition of the ex-food products used, representing a critical issue for their inclusion in balanced animal diets. Therefore, before incorporating a specific PBPI into a balanced diet as a partial replacement of conventional feed ingredients, a thorough nutritional evaluation is essential, and the involvement of an intermediate processor of former food products is very important to standardize their composition and formulation for use as commercial feed (Pinotti and Dell’Orto, 2011, 2021; Srikanthithasan et al., 2024). Furthermore, the EU regulations require that mandatory declarations include starch, total sugars (calculated as sucrose), and crude fat (if > 5%), which are the main nutritional components of the PBPI (European Commission, 2022).

The inclusion of PBPI into poultry diets has been analyzed in several studies of laying hens (Olafadehan et al., 2010; Shafey et al., 2011; Rocha Gebert et al., 2020) and broilers (Adeyemo et al., 2013; Al-Sagan et al., 2021; Srikanthithasan et al., 2024) and offered promising results. Further investigations have thus been recommended to increase the body of knowledge on the effect of PBPI on egg and meat performance in relation to different variables, such as composition type, inclusion rate, and feeding period.

To contribute to the development of circular economy strategies aimed at improving poultry sustainability, the present study evaluated the effects of PBPI in the diets of laying hens on egg production performance, egg weight and egg parameters, and edible egg composition. We used a commercially produced PBPI called Primo®, a mixture of bakery by-products, former foodstuffs from pasta, cereals, and chocolate products, and agro-industrial by-products (Srikanthithasan et al., 2024). Primo® is manufactured according to EU Regulation 2022/11044 on the Catalogue of feed materials (European Commission, 2022).

## Materials and methods

### Ethical statements

Bird handling was performed in accordance with the principles outlined in the Guidelines for the Care and Use of Agricultural Animals in Research and Teaching (Tucker et al., 2020). The experimental protocol, including bird management, was approved by the Animal Welfare Committee (OPBA) of the University of Milan (Italy) with authorization n° 10_2024.

### Experimental design and bird management

The present study was carried out at the poultry facility of the Animal Production Centre of the University of Milan (Lodi, Italy). A total of 180 Lohmann Brown® laying hens were obtained from Alberici Farm (Castelletto Sopra Ticino, NO, Italy) and housed in furnished cages (Big Dutchman International GmbH, Vechta, Germany), according to Council Directive 1999/74/EC (European Union, 1999). Each cage measured 150 × 60 × 52 cm (w x d x h) and was equipped with automatic nipple drinkers (n. 4 nipples), linear feeders (12 cm/hen), plastic perches (15 cm/hen) and a nest box. An automatic belt system was present under the cage floor for manure removal. Eggs were manually collected from the egg belt located under the feeder line outside the cage. Hens were housed at 19 wk of age and reared for 16 wk. Hens were individually identified using removable leg rings and randomly assigned to three experimental treatments. Each experimental group was randomly allocated to five cages, with 12 hens/cage, providing 750 cm²/hen. Birds were given 14 days to adapt to their new environment following their arrival. The conditions in the facility were fully controlled and maintained at a temperature of 22–24°C, a relative humidity of 50–60%, and adequate air quality provided by a positive pressure forced ventilation system. The starting photoperiod was set at 10L:14D, which was increased by 1 h on a weekly basis until 14L:10D was reached at 23 wk of age. Mortality and health status were monitored and recorded twice daily.

### Experimental diets

During the 14-day adaptation period, all hens were fed a standard commercial diet for pre-oviposition (CP 17.1%, EE 4%, Calcium 2.1%). From 21 to 35 weeks of age, the three groups received the following dietary treatments: (1) a standard control diet (CD) for laying hens in oviposition; (2) CD with 20% (PBPI20) Primo® product inclusion; (3) CD with 40% (PBPI40) Primo® product inclusion. The Primo® product was provided by Dalma Mangimi SPA (Marene, CN, Italy) and its chemical composition is reported in Table 1. The Primo® product is a standardized mixture of selected former foodstuffs and agro-industrial by-products derived mainly from bakery items (e.g., bread, biscuits, cakes, and snacks), pasta, cereals, and chocolate products. The materials originated from large-scale retail and pre-sale production chains in compliance with current EU regulations.

**Table 1:** Chemical composition (%) of Primo® product.

Calculated contents	% as fed
DM	88.00
CP	10.50
Ether extract	10.50
Crude fiber	2.20
Nitrogen free extract	62.60
Ash	2.20
Starch	40.00
Total sugars ^1^	17.00
Sodium	0.30
Calcium	0.10
Phosphorus	0.18
Potassium	0.39
Chloride	0.29
*Amino acids, % in CP*	
Lysine	0.28
Cystine	0.22
Phenylalanine	0.52
Isoleucine	0.40
Leucine	0.79
Methionine	0.17
Valine	0.48
Tyrosine	0.32
Proline	1.07
Alanine	0.37
Threonine	0.32
Arginine	0.46
Histidine	0.24
Glycine	0.39
Serine	0.51
Glutamic acid	3.30
Aspartic acid	0.55
*Fatty acids, % in total FA^2^*	
ƩSFA^3^	40.21
ƩMUFA^4^	45.17
ƩPUFA^5^	14.62

^1^Expressed in sucrose

^2^ FA: fatty acids

^3^ƩSFA: total saturated fatty acids

^4^ƩMUFA: total monounsaturated fatty acids

^5^ƩPUFA: total polyunsaturated fatty acids.

The diets were isoenergetic, isonitrogenous, and contained the same proportion of linoleic acid. The ingredients and chemical composition of the experimental diets are reported in Table 2. The diets were provided in crumbled form and offered *ad libitum* once a day. Fresh drinking water was always available.

**Table 2:** The ingredients, chemical and fatty acid composition of the experimental diets.

Calculated contents	CD^1^	PBPI20^1^	PBPI40^1^
*Ingredients* (g/kg as fed)			
Corn meal	496.6	293.6	147.1
Soybean meal	197.0	159.6	162.3
Primo®	0.0	200.0	400.0
Wheat	80.0	80.0	80.0
Sunflower protein	50.0	50.0	10.4
Wheat middlings	0.0	50.0	50.0
Bran	7.9	30.9	40.0
Alfalfa meal	12.2	1.5	0.0
Animal fat	43.3	29.4	1.8
Soybean oil	0.0	2.6	6.8
Calcium carbonate	88.7	89.7	89.9
Dicalcium phosphate	3.2	2.1	1.8
Sodium chloride	2.5	1.3	0.0
Sodium bicarbonate	0.8	0.5	0.5
DL-Methionine	1.7	1.7	1.9
L-Lysine	0.3	1.1	1.3
Tryptophan	0.0	0.2	0.4
Choline chloride	1.2	1.2	1.2
Farmicol 5R-rox R 50^2^	0.8	0.8	0.8
BELFEED B220^3^	0.5	0.5	0.5
PBA HIPHOS ^4^	2.0	2.0	2.0
Mineral-vitamin premix^5^	1.3	1.3	1.3
*Chemical composition (% as fed, analyzed)*			
DM	92.48	92.31	92.19
CP	16.71	16.69	16.64
Ether extract	5.99	6.05	6.11
Crude fiber	5.01	5.07	4.57
Ash	10.49	10.94	10.77
C18:2n-6 (%)	1.60	1.60	1.60
Total sugar (expressed in sucrose, % as fed DM)	2.62	5.71	8.81
Total xanthophylls (mg/kg)	16.78	11.38	7.68
*Fatty acid composition (% of total FA^2^, analyzed)*			
Σ SFA^6^	35.52	32.32	32.52
C16:0	22.31	21.13	20.23
C18:0	10.78	8.49	8.97
Σ MUFA^6^	35.83	34.09	38.97
C18:1n-9	33.08	31.85	37.32
Σ PUFA^6^	29.58	34.52	28.51
C18:2n-6	27.72	31.96	26.40
C18:3n-3	1.65	2.39	2.02
PUFA/SFA	0.83	1.07	0.88
Σn-6/n-3	16.92	13.46	13.08

^1^CD: standard control diet; PBPI20: CD with 20% Primo® product inclusion; PBPI40: CD with 40% Primo® product inclusion

^2^Farmicol 5R-rox R 50: xanthophylls

^3^BELFEED B220: xylanase

^4^PBA HIPHOS: phytase IT200001MI Ricon. Reg. (CE) n°183/2005

^5^Mineral-vitamin premix: vitamin A, 8′000′000 I.U.; vitamin D3, 2′400′000 I.U.; vitamin E, 16′000 mg; vitamin K3, 800 mg; vitamin B1, 320 mg; vitamin B2, 2′040 mg; vitamin mg, 1′200 I.U.; vitamin B12, 12 mg; pantothenate, 4′800 mg; niacin, 12′000 mg; folic acid, 240 mg; Fe, 15′200 mg; Mg, 50′000; Zn, 40′000; I, 640 mg; Se, 240 mg; Cu, 6′400 mg

^6^ƩSFA: total saturated fatty acids; ƩMUFA: total monounsaturated fatty acids; ƩPUFA: total polyunsaturated fatty acids

### Body weight and egg production

Hen body weight (BW) (kg) was recorded at 21 wk of age and every two wk thereafter (23, 25, 27, 29, 31, 33, 35 wk of age).

The feed provided to each cage of hens was weighed daily and any residual feed was weighed every two wk so as to calculate the average daily feed intake (ADFI) (g/d), where: ADFI = (weight of feed provided per cage in 14 d – weight of residual feed) / 14 days / 12 hens.

Eggs were collected daily. The number of eggs/cage (n), the total egg mass/cage (EM, kg), the oviposition percentage (%) were calculated every two weeks. The feed conversion ratio was calculated per egg mass unit (FCRemu) and per dozen eggs (FCRde). We used the net feed efficiency index (NFEI), based on EM, BW, and feed consumption during a specific period as a proxy of the hens’ production efficiency. NFEI was calculated using the formula: NFEI = (EM + BW) * 100 / ADFI. A score of 45 or higher was considered desirable (Narahari et al., 1983).

### Egg physical analyses

A sample of 20 eggs/treatment (4 eggs/cage) was collected from each experimental group every two wk, specifically at 23, 25, 27, 29, 31, 33, and 35 wk of age. Eggs were stored at +4°C for 5 d and warmed at 18°C for 4 h before analyses.

Egg weight (EW, g) was measured using a digital precision scale (GP3202, Sartorius Srl, MB, Italy). Egg diameter (B, equatorial axis) and length (L) were recorded using a micrometer (Digital Stainless Hardened, France), and the shape index (SI) was calculated as B/L*100 (Duman et al., 2016). We calculated egg volume (EV=0.525*L* B²; cm³), surface area (SA= [0.9658 * B/L] + [2.1378 * L * B]; cm²), and SA/EV ratio (Narushin, 2005). We assessed eggshell color using a Chroma Meter (Minolta CR-300, Singapore, Japan) and the CIE-Lab system for values of lightness (L∗), redness (a∗), and yellowness (b∗) (CIE, 1986).

We assessed the following internal physical egg parameters: yolk weight (YW, g), shell thickness (ST, mm), and eggshell weight (SW, g) following cleaning with demineralized water and drying in an oven at 45°C for 12 h (Rizzi et al., 2022). We calculated albumen weight (AW, g) as EW – (YW + SW). Yolk color was assessed using a Chroma Meter (Minolta CR-300, Singapore, Japan) and the CIE-Lab system for values of lightness (L∗), redness (a∗), and yellowness (b∗) (CIE, 1986), and also using the DSM YolkFan™ method (DSM, Heerlen, the Netherlands). We calculated the following proportions (%): SW/EW, YW/EW, AW/EW, (YW+AW)/EW, YW/AW.

Finally, the edible components (yolk + albumen) of the eggs (n. 4) collected from a single cage were pooled and homogenized for chemical and fatty acid analyses. Five pools (1 pool/cage) were analyzed per treatment at 23, 29, 35 wk of age. Pooled egg samples were stored at -20°C until further analysis.

### Egg chemical analyses

The following chemical components were analyzed in duplicate in edible egg samples: DM (EU 152/2009), CP (2001.11; AOAC, 2005), ether extract (EE) (GU n. 31 08/02/1999, DM 21/12/1998) and ash (942.05; AOAC, 2005) (AOAC, 2005; European Commission, 2009).

### Egg fatty acid composition

Total lipids (TL) were extracted and quantified according to the Folch method (Folch et al., 1957). To determine their fatty acid composition, we used gas chromatography following their conversion into fatty acid methyl esters (FAMEs) according to the procedure described by Christie (2003). In brief, an aliquot of 20 mg TL was dried under a gentle stream of nitrogen, and 1 mL of tricosanoic acid (C23:0) in toluene was added as internal standard. Then, 2 mL of an acetyl chloride mixture in methanol (1:10, v/v) was added to each sample and the reaction carried out at 50°C in sealed tubes overnight. After cooling, 2 mL of a 1 M potassium carbonate solution and 5 mL of 5% sodium chloride were added to each tube and the resulting FAMEs extracted using hexane (2 × 2 mL). The organic phase containing the FAMEs was dried under a gentle stream of nitrogen and the final residue reconstituted in 1 mL of hexane. FAME separation was performed using a gas chromatograph (TRACE^TM^ 1300, Thermo Fischer Scientific, USA) equipped with a split-splitless injector (split ratio 1:100), a capillary column (TRACE™ TR-FAME 30 m x 0.25 mm x 0.25 μm, Thermo Fischer Scientific, USA) and a flame ionization detector. Fatty acids were identified through the comparison of retention times with Supelco® 37 component FAME mix standards (Sigma-Aldrich S.r.l., Milan, Italy) and expressed as a percentage of total fatty acids.

### Statistical analysis

Data were analyzed using SAS software, version 9.4 for Windows (SAS Institute, 2025).

ANOVA was performed on all data related to feed consumption, egg production performance and egg quality; the statistical model included dietary treatments, the week of age, and their interaction as sources of variation; the cage was considered the experimental unit. Least-square means (LSMeans) were compared using Student’s *t* tests, and significance was set at *P* < 0.05.

ANOVA for repeated measures was performed on BW data. The dietary treatment, the wk of age and the related interaction were considered as fixed effects, and the hen as a random effect. LSMeans were compared using Student’s *t* test and significance was set at *P* < 0.05.

## Results

### Body weight and egg production

Hen BW (kg) was not significantly affected by the dietary treatments or by any diet*age interaction. As expected, BW was significantly affected by hen age (*P =* 0.001). A significant increase in BW occurred over the experimental period (21–35 weeks of hen age), rising from 1.86 kg at 21 weeks to 1.99 kg at 35 weeks of age (*P* < 0.05); the mean BW for the entire period was 1.96 kg.

The results on egg production parameters are presented in Table 3. The diet significantly affected EM, ADFI, FCRemu, FCRde, and NFEI. Bird age significantly affected all egg production parameters, except FCRemu, and there was no significant diet*age interaction for any of the egg parameters (Table 3). The mean number of eggs biweekly produced by the three treatment groups was 157, with a mean oviposition rate of 93.4% and no significant differences between dietary treatments. By contrast, the mean EM produced by laying hens fed the PBPI40 was significantly lower compared with that for hens fed CD and PBPI20, which showed similar values (9.48 kg *vs* 9.79 kg and 9.87 kg, respectively; *P* < 0.05).

**Table 3:** Egg production parameters recorded in laying hens fed the control (CD), 20% (PBPI20) and 40% (PBPI40) Primo® supplemented diets from 23 to 35 wk of age.

	Egg (n)	Egg mass (kg)	Oviposition (%)	ADFI (g/d)	FCRemu^1^	FCRde^2^	NFEI^3^
Dietary treatments							
CD	157.03±1.15	9.79±0.07^a^	93.91±0.58	111.59±1.27^a^	1.89±0.02^b^	1.41±0.02^b^	56.93±0.59^a^
PBPI20	157.34±1.15	9.87±0.07^a^	93.66±0.58	119.43±1.27^b^	2.01±0.02^a^	1.51±0.02^a^	53.97±0.59^b^
PBPI40	155.48±1.15	9.48±0.07^b^	92.57±0.58	116.36±1.27^b^	2.04±0.02^a^	1.49±0.02^a^	54.10±0.59^b^
Wk of age							
23	145.27±1.51^z^	8.37±0.1^z^	86.47±0.85^z^	103.64±1.94^y^	1.93±0.03	1.34±0.03^w^	58.28±1.02^v^
25	164.13±1.51^v^	9.91±0.1^vwx^	97.70±0.85^v^	120.70±1.94^vw^	2.04±0.03	1.48±0.03^v^	53.83±1.02^w^
27	160.00±1.51^vw^	10.06±0.1^vwx^	95.24±0.85^wx^	120.12±1.94^vw^	2.01±0.03	1.52±0.03^v^	53.30±1.02^w^
29	162.00±1.51^v^	10.19±0.1^vw^	96.43±0.85^vw^	121.10±1.94^v^	2.00±0.03	1.51±0.03^v^	54.33±1.02^w^
31	157.60±1.51^wx^	9.97±0.1^vx^	93.81±0.85^xy^	116.32±1.94^vwx^	1.96±0.03	1.49±0.03^v^	55.30±1.02^w^
33	154.40±1.51^xy^	9.87±0.1^xy^	91.90±0.85^y^	115.52±1.94^wx^	1.95±0.03	1.50±0.03^v^	54.54±1.02^w^
35	152.93±1.51^y^	9.62±0.1^y^	92.09±0.85^y^	113.16±1.94^x^	1.97±0.03	1.49±0.03^v^	55.42±1.02^w^
Source of variation		*P values*					
Diet	ns	0.0001	ns	0.0002	<.0001	0.0002	0.0030
Age	<.0001	<.0001	<.0001	<.0001	ns	0.0001	0.0255
Diet*age	ns	ns	ns	ns	ns	ns	ns

^1^FCRemu: feed conversion/egg mass ratio

^2^FCRde: feed conversion/dozen egg ratio

^3^NFEI: net feed efficiency index

^a,b^different superscript letters indicate significant differences between experimental dietary treatments (*P*<0.05)

^v,w,x,y,z^different superscript letters indicate significant differences between ages (*P*<0.05)

The mean values of ADFI (g/d), FCRemu and FCRde were similar in the PBPI20 and PBPI40 groups, and both were significantly higher compared with the CD group (*P <* 0.05). An opposite trend was found for NFEI, which was significantly lower in two experimental groups than in CD (*P* < 0.05); however, NFEI mean values recorded in all treatment groups were much higher compared with the threshold of 45 considered to be desirable (Table 3).

Egg production, EM, oviposition rate, and ADFI showed a similar trend during the experimental period, characterized by an initial significant increase between 23 and 29 wk of age (*P* < 0.05) followed by a significant decrease until 35 wk of age (*P* < 0.05). Statistical analysis revealed significant differences between several ages (Table 3).

### Egg physical analyses

ANOVA results on EW, YW, AW, SW, YW/AW ratio and the relative mean values are presented in Table 4. A significant effect of dietary treatment was observed for SW only (*P* = 0.0251), whereas age significantly affected all the physical parameters of the eggs produced (*P* < 0.001). The results revealed no significant interaction of diet*age.

**Table 4:** Egg physical parameters recorded in laying hens fed the control (CD), 20% (PBPI20) and 40% (PBPI40) Primo® supplemented diets from 23 to 35 wk of age.

	Egg (g)	Shell (g)	Albumen (g)	Yolk (g)	Shell (%)	Albumen (%)	Yolk (%)	YW/AW^1^
Dietary treatments								
CD	62.80±0.39	7.42±0.12^a^	39.19±0.34	16.19±0.13	12.08±0.25	62.16±0.29	25.73±0.21	0.42±0.004
PBPI20	62.85±0.39	7.35±0.12^a^	39.14±0.33	16.36±0.12	11.71±0.25	62.19±0.29	26.09±0.21	0.42±0.004
PBPI40	62.06±0.38	6.98±0.12^b^	38.80±0.33	16.27±0.12	11.27±0.25	62.47±0.29	26.26±0.21	0.42±0.004
Weeks of age								
23	59.06±0.61^w^	7.50±0.19^wy^	37.68±0.53^w^	13.92±0.20^z^	12.68±0.39^vx^	63.71±0.46^v^	23.60±0.33^x^	0.37±0.007^y^
25	63.80±0.59^v^	8.15±0.18^v^	40.20±0.51^v^	15.45±0.19^y^	12.80±0.38^v^	62.92±0.44^v^	24.28±0.32^x^	0.39±0.007^xy^
27	63.07±0.59^v^	7.55±0.18^wy^	39.87±0.51^v^	15.65±0.19^y^	11.96±0.38^vx^	63.10±0.44^v^	24.94±0.32^x^	0.40±0.007^x^
29	62.22±0.59^v^	7.65±0.18^vw^	38.22±0.51^v^	16.35±0.19^x^	12.33±0.38^v^	61.33±0.44^w^	26.34±0.32^w^	0.43±0.007^w^
31	63.03±0.60^v^	6.29±0.19^x^	39.79±0.52^v^	16.95±0.19^w^	10.59±0.38^w^	62.53±0.45^w^	26.72±0.32^w^	0.43±0.007^w^
33	63.25±0.59^v^	6.53±0.18^x^	39.55±0.51^v^	17.16±0.19^w^	10.32±0.38^w^	62.47±0.44^w^	27.21±0.32^w^	0.44±0.007^w^
35	63.52±0.59^v^	7.08±0.18^y^	38.02±0.51^w^	18.42±0.19^v^	11.14±0.38^wx^	59.77±0.44^x^	29.08±0.32^v^	0.49±0.007^v^
Source of variation	*P values*							
Diet	ns	0.0251	ns	ns	ns	ns	ns	ns
Age	<.0001	<.0001	0.0006	<.0001	<.0001	<.0001	<.0001	<.0001
Diet*age	ns	ns	ns	ns	ns	ns	ns	ns

1 YW/AW: yolk weight / albumen weight ^a,b^different superscript letters indicate significant differences between experimental dietary treatments (*P*<0.05) ^v,w,x,y,z^different superscript letters indicate significant differences between ages (*P*<0.05)

No significant differences were found between dietary treatments with regard to the mean values of EW, AW, or YW, albumen and yolk proportions, or YW/AW (Table 4). The overall means were 62.57 g, 39.04 g, and 16.27 g, for EW, AW, and YW, respectively, and 11.69%, 62.27%, 26.03%, and 0.42 for shell, albumen, and yolk proportions, and YW/AW, respectively.

In contrast, the mean SW recorded in the PBPI40 group was significantly lower compared with the mean values recorded in CD and PBPI20 (6.98 g *vs* 7.42 g and 7.35 g, respectively, *P* < 0.05); however, no significant differences were observed between treatments in shell proportion (Table 4).

Mean EW significantly increased from 59 g to 64 g from 23 to 25 wk of age (*P* < 0.05), but no further significant differences were found thereafter. Mean SW showed significant differences between several ages; however, a clear trend was not identified, and similar mean values were recorded at the beginning and end of the experimental period (23 and 35 wk of age, respectively) (Table 4). Despite the few differences between ages, mean AW did not significantly change from 23 to 35 wk of age, whereas mean YW showed a significant progressive increase across the experimental period, from 13.92 g at 23 wk of age to 18.42 g at 35 wk of age (*P* < 0.05). The proportional composition of the egg also changed over the course of the experimental period. In particular, the shell and albumen proportions significantly decreased, whereas the yolk proportion and the YW/AW ratio significantly increased (Table 4).

ANOVA on the SA, EV, and SI of the eggs produced showed no significant differences between dietary treatments, hen age, and their interaction (data not presented). The overall mean values for SA, EV, and SI recorded during the experimental period were 73.06 cm^2^, 59.13 cm^3^, and 78.5, respectively.

The ANOVA results for ST, shell and yolk color, and the relative mean values are presented in Table 5. The dietary treatments had a significant effect on both shell and yolk color, and age had a significant effect on ST, shell and yolk color. There was no significant diet*age interaction for any of these parameters. The mean values of a* and b* for shell color recorded in the PBPI40 group were significantly lower compared with the values for CD and PBPI20 (a*: 14.09 *vs* 15.78 and 15.77, *P* < 0.05; b*: 28.74 *vs* 30.05 and 29.73, *P* < 0.05), whereas the opposite trend was found for the L* (58.36 *vs* 56.13 and 56.39, *P* < 0.05). Also in the yolk, L* was significantly higher in PBPI40 and PBPI20 than in CD (49.19 and 49.47 *vs* 48.03, *P <* 0.05), and the b* was significantly lower in PBPI40 compared with the control values (32.45 *vs* 34.54, *P* < 0.05) (Table 5). In contrast, we recorded very similar mean values of ST, the yolk FAN score, and yolk a* across all treatment groups. ST and yellowness (b*) significantly decreased from 0.39 mm and 32.21, respectively, at 23 wk of age to 0.36 mm and 29.65, respectively, at 35 wk of age (*P* < 0.05). By contrast, the yolk L* and b* showed a significant progressive increase across the experimental period, rising from 42.21 and 31.0, respectively, at 23 wk of age to 51.55 and 37.77, respectively, at 35 wk of age (P < 0.05) (Table 5).

**Table 5:** Shell thickness (ST), shell and yolk color recorded in eggs from laying hens fed the control (CD), 20% (PBPI20) and 40% (PBPI40) Primo® supplemented diets from 23 to 35 wk of age.

	ST (mm)	Shell L*	Shell a*	Shell b*	Yolk color^1^	Yolk L*	Yolk a*	Yolk b*
Dietary treatments								
CD	0.37±0.005	56.13±0.35^b^	15.78±0.21^a^	30.05±0.22^a^	12.29±0.07	48.03±0.40^b^	6.03±0.21	34.54±0.38^a^
PBPI20	0.37±0.005	56.39±0.34^b^	15.77±0.21^a^	29.73±0.22^a^	12.09±0.07	49.47±0.39^a^	5.76±0.20	33.88±0.38^a^
PBPI40	0.36±0.005	58.36±0.34^a^	14.09±0.21^b^	28.74±0.22^b^	12.28±0.07	49.19±0.39^a^	6.30±0.20	32.45±0.38^b^
Weeks of age								
23	0.39±0.01^v^	57.19±0.54	16.22±0.33	32.21±0.35^v^	12.06±0.09	42.21±0.62^z^	6.46±0.32	31.70±0.60^z^
25	0.35±0.01^w^	46.78±0.52	16.99±0.32	32.36±0.34^v^	12.13±0.09	48.48±0.60^yx^	6.72±0.32	35.52±0.57^w^
27	0.39±0.01^v^	57.77±0.52	16.81±0.32	30.74±0.34^w^	12.02±0.09	49.99±0.60^yw^	6.81±0.31	29.84±0.57^x^
29	0.35±0.01^w^	57.62±0.52	16.25±0.32	30.53±0.34^w^	12.05±0.09	47.00±0.60^x^	6.64±0.31	27.16±0.58^y^
31	0.35±0.01^w^	55.71±0.53	17.08±0.32	31.11±0.34^w^	12.02±0.09	50.28±0.61^w^	6.74±0.32	37.34±0.58^v^
33	0.35±0.01^w^	56.57±0.52	16.71±0.32	29.90±0.34^x^	12.07±0.09	52.77±0.60^v^	6.55±0.31	36.01±0.57^wv^
35	0.36±0.01^w^	57.07±0.52	16.41±0.32	29.65±0.34^x^	11.95±0.09	51.55±0.60^vw^	6.80±0.31	37.77±0.57^v^
Source of variation				*P* values				
Diet	ns	<.0001	<.0001	0.0001	ns	0.0266	ns	0.0004
Age	<.0001	ns	ns	<.0001	ns	<.0001	ns	<.0001
Diet*age	ns	ns	ns	ns	ns	ns	ns	ns

^1^DSM YolkFan™ method

^a,b^different superscript letters indicate significant differences between experimental dietary treatments (*P*<0.05)

^v,w,x,y,z^different superscript letters indicate significant differences between ages (*P*<0.05)

### Egg chemical analyses

ANOVA revealed dietary treatment to have a significant effect on only the ash content of the edible egg component, whereas hen age significantly affected all chemical components (DM, EE, CP, and ash), and there was no significant effect of diet*age interaction (Table 6). The mean chemical composition of all eggs analyzed was: 25.3% DM, 12.6% CP, 8.1% EE, and 1.1% ash. The ash content recorded in the CD group was significantly higher than that for PBPI40 (1.28% *vs* 0.97%; *P <* 0.05), and an intermediate mean value was found for PBPI20 (1.20%). The DM, EE, and ash content of edible eggs was significantly greater at 29 wk of age compared with 23 and 25 wk of age, whereas an opposite trend was found for CP content (Table 6).

**Table 6:** Chemical composition (%) of eggs from laying hens fed the control (CD), 20% (PBPI20) and 40% (PBPI40) Primo® supplemented diets from 23 to 35 wk of age.

	DM	CP	Ether extract	Ash
Dietary treatments				
CD	25.37 ± 0.60	12.65 ± 0.10	8.14 ± 0.23	1.28 ± 0.08^a^
PBPI20	25.41 ± 0.60	12.67 ± 0.10	8.53 ± 0.23	1.20 ± 0.08^ab^
PBPI40	24.73 ± 0.60	12.59 ± 0.10	7.71 ± 0.23	0.97 ± 0.08^b^
Weeks of age				
23	23.88 ± 0.6^w^	12.75 ± 0.10^v^	7.25 ± 0.23^w^	1.02 ± 0.08^w^
29	24.31 ± 0.60^w^	12.84 ± 0.10^v^	7.84 ± 0.23^w^	1.04 ± 0.08^w^
35	27.32 ± 0.60^v^	12.43 ± 0.10^w^	9.28±0.23^v^	1.39 ± 0.08^v^
Source of variation	*P* values			
Diet	ns	ns	ns	0.0321
Age	0.0004	0.0217	<.0001	0.0047
Diet*age	ns	ns	ns	ns

^a,b^different superscript letters indicate significant differences between experimental dietary treatments (*P*<0.05)

^v,w^different superscript letters indicate significant differences between ages (*P*<0.05)

### Egg fatty acid composition

The results of ANOVA on the fatty acid composition of eggs and on the proportions of SFAs, MUFAs, PUFAs, n-6, n-3, and the n-6/n-3 ratio are presented in Table 7, Table 8. Overall, the analysis revealed MUFAs (mainly represented by C18:1n-9) to be the predominant fraction (47.1%), followed by SFAs (34.5%). The proportion of PUFAs (13.6%) mainly consisted of n-6 series FAs, primarily C18:2n-6.

**Table 7:** Fatty acid composition (%) of eggs from laying hens fed the control (CD), 20% (PBPI20) and 40% (PBPI40) Primo® supplemented diets from 23 to 35 wk of age (fatty acids > 0.4% are reported).

	C14:0	C16:0	C18:0	C16:1	C18:1n9	C18:2n6	C18:3n3	C20:4n6	C22:6n3
Dietary treatments									
CD	0.45 ± 0.01	24.79 ± 0.13^a^	9.24 ± 0.24	2.56 ± 0.08^a^	44.03 ± 0.52	13.87 ± 0.32	0.37 ± 0.01^b^	2.27 ± 0.13	0.84 ± 0.06
PBPI20	0.44 ± 0.01	24.04 ± 0.14 ^b^	9.15 ± 0.16	2.30 ± 0.06^b^	44.67 ± 0.45	14.54 ± 0.29	0.43 ± 0.01^a^	2.20 ± 0.11	0.85 ± 0.04
PBPI40	0.46 ± 0.01	24.41 ± 0.18 ^ab^	8.62 ± 0.33	2.37 ± 0.10^ab^	45.16 ± 0.45	14.24 ± 0.46	0.41 ± 0.02^ab^	2.15 ± 0.04	0.82 ± 0.03
Weeks of age									
23	0.42 ± 0.01^w^	24.66 ± 0.14^v^	9.24 ± 0.42	2.59 ± 0.08^v^	44.47 ± 0.70	13.52 ± 0.28^w^	0.37 ± 0.01^w^	2.42 ± 0.15	0.91 ± 0.06
29	0.44 ± 0.01^w^	24.77 ± 0.12^v^	9.09 ± 0.14	2.46 ± 0.05^v^	45.00 ± 0.43	13.46 ± 0.27^w^	0.38 ± 0.01^w^	2.16 ± 0.06	0.83 ± 0.03
35	0.48 ± 0.0^v^	23.82 ± 0.12^w^	8.69 ± 0.05	2.18 ± 0.08^w^	44.38 ± 0.18	15.68 ± 0.18^v^	0.46 ± 0.01^v^	2.03 ± 0.02	0.79 ± 0.02
Source of variation	*P values*								
Diet	ns	0.003	ns	0.031	ns	ns	0.002	ns	ns
Age	<.001	<.001	ns	<.001	ns	<.001	<.001	ns	ns
Diet*age	0.005	ns	ns	0.001	<.001	<.001	0.018	ns	ns

^a,b^different superscript letters indicate statistically significant differences among the three groups based on diet (*P*<0.05)

^v,w^different superscript letters indicate statistically significant differences among the three groups based on age (*P*<0.05)

**Table 8:** Total saturates (SFAs), monounsaturates (MUFAs), polyunsaturates (PUFAs), n-6 and n-3 and n-6/n-3 in eggs from laying hens fed the control (CD), 20% (PBPI20), and 40% (PBPI40) Primo® supplemented diets from 23 to 35 wk of age.

	SFAs (%)	MUFAs (%)	PUFAs (%)	n-6 (%)	n-3 (%)	n-6/n-3
Dietary treatments						
CD	35.23 ± 0.34^a^	47.11 ± 0.53	17.66 ± 0.38	16.45 ± 0.36	1.21 ± 0.05	13.83 ± 0.47
PBPI20	34.23 ± 0.24^b^	47.44 ± 0.44	18.33 ± 0.34	17.05 ± 0.32	1.28 ± 0.04	13.42 ± 0.26
PBPI40	34.09 ± 0.33^b^	47.97 ± 0.50	17.94 ± 0.49	16.71 ± 0.46	1.23 ± 0.04	13.62 ± 0.35
Weeks of age						
23	34.89 ± 0.42^v^	47.59 ± 0.72^v^	17.52 ± 0.43^w^	16.25 ± 0.38^w^	1.27 ± 0.06	13.03 ± 0.36^w^
29	35.02 ± 0.26^v^	47.85 ± 0.41^v^	17.12 ± 0.30^w^	15.92 ± 0.29^w^	1.21 ± 0.04	13.32 ± 0.39^w^
35	33.64 ± 0.11^w^	47.08 ± 0.18^w^	19.29 ± 0.21^v^	18.04 ± 0.18^v^	1.25 ± 0.03	14.51 ± 0.23^v^
Source of variation	*P values*					
Diet	0.029	ns	ns	ns	ns	ns
Age	<.001	0.029	<.001	<.001	ns	0.003
Diet*age	ns	<.001	<.001	<.001	ns	0.002

^a,b^different superscript letters indicate statistically significant differences among the three groups based on diet (*P*<0.05)

^v,w^different superscript letters indicate statistically significant differences among the three groups based on age (*P*<0.05)

^a,b^different letters indicate significant difference between treatments (*P*<0.05)

Significant differences were found in FA composition between dietary treatments, specifically for C16:0, C16:1, and C18:3n-3; and between hen ages, specifically for C14:0, C16:0, C16:1, C18:2n-6, and C18:3n-3. Significant diet*age interactions were found involving C14:0, C16:1, C18:1n-9, C18:2n-6, and C18:3n-3 (Table 7). In relation to the dietary treatment, C16:0 and C16:1 were significantly higher in CD compared with PBPI20 (24.79% *vs* 24.04%, *P* < 0.05 for C16:0; 2.56% *vs* 2.30%, *P* < 0.05 for C16:1), whereas PBPI40 showed intermediate values (C16:0=24.41%; C16:1=2.37%). Conversely, C18:3n-3 was significantly higher in PBPI20 compared with CD (*P* < 0.05), and PBPI40 displayed an intermediate value. At 35 wk of age, edible eggs showed significantly higher proportions of C18:2n-6 and C18:3n-3 and a concomitantly smaller proportion of C14:0, C16:0, and C16:1 than at 23 and 29 wk of age (Table 7).

According to the ANOVA results, SFAs were significantly affected by both diet and age factors, whereas MUFAs, PUFAs, total n-6, and the n-6/n-3 ratio were significantly affected by age and showed a diet*age interaction (Table 8). A significantly higher proportion of SFA was found in the CD group compared with both experimental groups; furthermore, the SFA proportion significantly increased from 23 and 29 wk to 35 wk of age (Table 8).

**Fig. 1:**
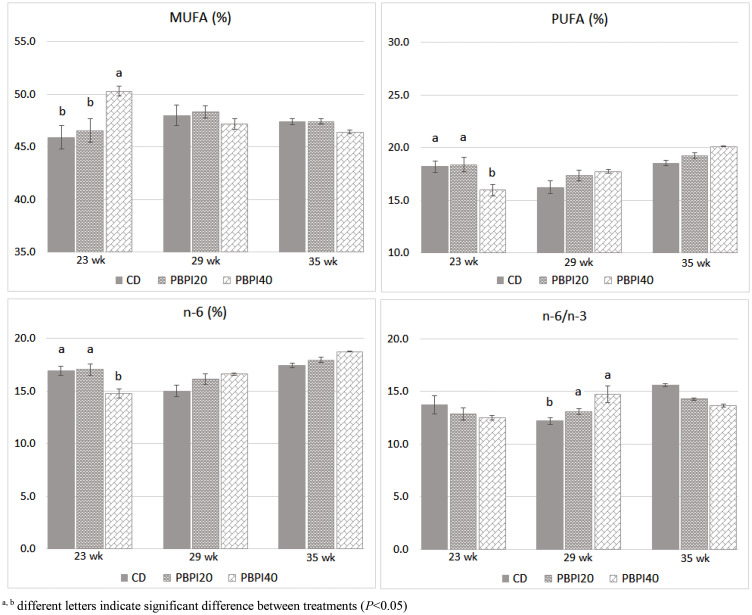
Total monounsaturates (MUFA), polyunsaturates (PUFA) and n-6, and n-6/n-3 ratio in eggs from laying hens fed the control (CD), 20% (PBPI20) and 40% (PBPI40) Primo® supplemented diets at 23, 29, and 35 wk of age.

The MUFA proportion significantly decreased at 35 wk of age. While the three dietary treatments showed similar mean values for the proportion of MUFAs at 29 and 35 wk of age, at the youngest age (23 weeks) it was significantly higher in hens fed the PBPI40 diet (Fig. 1). The PUFA and n-6 proportions were significantly lower in PBPI40 eggs at 23 wk of age compared with eggs from CD and PBPI20, and no further differences between treatments were found at the older ages. The n-6/n-3 ratio was similar across all dietary treatments at 23 and 35 wk of age, whereas significantly higher values were found at 29 wk of age in both PBPI treatment groups compared with eggs from hens receiving the CD (Fig. 1).

## Discussion

The present study evaluated the egg production efficiency of laying hens fed PBPI for 14 wk from the beginning of the laying cycle, along with the physical and chemical quality of eggs. The use of PBPI in the hen diet composition and the inclusion level affected several production and egg-quality parameters. The number of eggs laid, and the oviposition rate were not affected by the inclusion of PBPI. By contrast, EM was negatively affected only at the higher inclusion level (40%), probably dependent by a concomitant slight decrease in the number and weight of eggs. These results are consistent with previous studies reporting no differences in the oviposition rate of layers fed 20% bread waste (Grigorova and Penkov, 2023) or a wide range (20 to 100%) of extruded bakery waste as a replacement for maize (Shafey et al., 2011). Similar to the present study, these Authors also found no differences in EM. By contrast, feeding Isabrown layers a diet supplemented with 20 or 30% bakery waste products from 35 wk of age for 8 wk improved the oviposition rate from 70 to 75–76% (Olafadehan et al., 2010). These discordant results may be related to the different origins and preparation of the ex-food products used, which have the potential to influence the nutritional composition of the diet, its nutrient digestibility and then productive performances (Luciano et al., 2020). The period of dietary supplementation during the hen laying cycle, characterized by specific productive trends and related nutrient requirements, might also modulate the potential effect of PBPI.

The ADFI of the layers was positively affected by feeding the Primo® product at both levels of dietary inclusion. The Primo® product is characterized by a high content of starches and sugars and a distinctive aroma, all of which might have improved the diet’s palatability and led to an increase in feed intake. By contrast, dietary supplementation with bread (Shafey et al., 2011; Grigorova and Penkov, 2023) and bakery waste products (Olafadehan et al., 2010) were reported as having no effect on layer feed intake. The different sources of PBPI used in the present study might account for this discrepancy; in fact, products obtained only or mainly from bread waste would be expected to differ significantly from the Primo® product derived from confectionery industry waste. As a consequence of the higher feed intake of layers fed the Primo® product, both FCRemu and FCRde were greater in the experimental groups compared with the control group. Despite the higher ADFI, no variations in BW were observed between dietary treatments throughout the experimental period, suggesting that the larger amounts of feed, and thus nutrients consumed were not used to support body development.

Physical and chemical egg traits were partially affected by the inclusion of PBPI in the hens’ diet. A 20% rate of PBPI inclusion did not change egg quality, with similar physical and chemical traits found in both in the PBPI20 and in the control group; the only exception was the yolk color lightness index (L*), which was higher in the PBPI20 group, indicating a brighter yolk color in these eggs. Similar results were reported in hens fed bread waste products, which also produced eggs characterized by several similar physical traits (EW, YW, AW and AW/EW proportion, SI and ST) and lighter colored yolk compared with control eggs (Grigorova and Penkov, 2023; Shafey et al., 2011). When the dietary inclusion of PBPI increased to 40%, further differences were found in egg quality compared with the control and PBPI20 groups. In particular, SW was lower in the PBPI40 group, and this reduction may be related to the higher sugar content in the diet fed the same experimental group. Specifically, high sugar levels can promote abnormal fermentation in the gut microbiota, which increases digesta viscosity and then alter nutrient utilization, particularly calcium and phosphorus. In addition, excess sugar metabolism creates an acidic environment in blood and intestines, altering the acid-base balance and impairing the transfer of bicarbonate ions, which are strictly required for calcification in the shell gland (Keshavarz and Austic, 1990; Svihus et al., 2013).

Furthermore, eggs from the PBPI40 group showed differences in eggshell and yolk colour intensity, characterized by higher L* and lower a* values in the eggshell, as well as lower b* values in both the yolk and eggshell. These colour variations may be related to the differences in diet composition, also involving the xanthophyll content. In fact, the level of xanthophyll was higher in the CD (16.78 mg/kg) than in PBPI20 and PBPI40 (11.38 and 7.68 mg/kg, respectively), due to the different dietary maize inclusion. Also, Miao et al. (2023) reported that yolk color, and in particular L* and b* values, can be modified by different dietary levels of carotenoids. Furthermore, the heat treatment applied during crumble feed production may have contributed to the additional reduction in yolk colour (Bozkurt et al., 2020). The variation in yolk color intensity, which might be received as undesirable in the market for table eggs, was detected by colorimetric assay, whereas the DSM YolkFan™ method did not reveal any perceived differences in yolk color between experimental groups. Therefore, the variation in yolk color detected in PBPI40 eggs by colorimetry is unlikely to be perceived by consumers and thus influence their purchasing preferences.

Dao et al. (2023) reported that feeding layers with a former food-based diet up to 34 wk of age was associated with a reduction in SW, ST, and eggshell proportion, whereas other internal and external egg quality traits were unaffected. The Authors suggested that the decrease in shell quality could be associated with the concomitant decrease in feed intake that might have resulted in lower calcium consumption or in an unbalanced calcium/mineral intake. In our study, feed intake was increased in layers fed PBPI compared with those receiving the control diet, meaning that a decrease in calcium consumption was unlikely to occur. The experimental diets, particularly the PBPI40 diet, contained higher amounts of bran than the CD. Bran is rich in insoluble fiber, which might reduce feed digestibility if present at concentrations that are too high or in an unprocessed form in which non-starch polysaccharides are not broken down (He et al., 2023).

The chemical composition of the edible egg portion corresponded to the standard composition of table eggs laid by a layer strain: 24–26% DM, 12–13% CP, 11–12% EE and 1–1.5% ash (Sauveur, 1988; Zaniboni and Cerolini, 2008). Neither of the two dietary inclusion rates of PBPI changed the nutrient composition of eggs, with the only exception of the ash content, which was reduced in the PBPI40 group, while the nutritional contents of eggs from CD and PBPI20 groups were the same. Similarly, Grigorova and Penkov (2023) reported no differences in the albumen and yolk compositions, including the ash content, of eggs from hens fed diets containing bread waste. Unfortunately, very few data are currently available on chemical analyses of table eggs from hens fed PBPI, thus, further studies are required to increase our knowledge on the effects of feeding PBPI on the nutrient composition of eggs.

Hen age had a significant effect on all the productive parameters, except FCRemu, and the dietary treatments did not affect the age-related changes. In our study, the number of eggs laid and the oviposition rate of Lohman layers increased significantly between 23 and 25 wk of age, reaching peak values between 25 and 29 wk, then gradually declining. This trend follows the standard performance of the commercial Lohman Brown laying strain (Lohmann Breeders, 2021). A similar trend was observed for EM, which progressively increased up to 29 wk of age, then showed a slight reduction. The present results are consistent with previous studies reporting a peak in egg production at 25–30 wk of age in ISA Brown layers (Anene et al., 2020) and at 25–28 wk of age in Shaver layers (Alig et al., 2025).

The ADFI progressively increased from 23 to 29 wk of age, with a slight decrease from thereafter until 35 wk of age. This pattern may be related to the high energy demand occurring during peak egg production and was consistent with the results found in ISA Brown layers, which showed an increasing ADFI during the early laying period up to the peak in production between 25 and 30 wk of age (Anene et al., 2020).With regard to the FA composition of eggs, the dietary treatments had very few effects. The PBPI20 diet had higher proportions of two essential FAs from the n-6 and n-3 series, namely, and C18:2n-6 and C18:3n-3. The higher concentration of the former was probably the reason for the higher concentration of this FA in eggs from the PBPI20 group, although its total lipid fraction remained very low, at only 0.43% of total FAs. Eggs from hens fed the CD showed higher overall levels of SFAs, likely reflecting the higher SFA content of the CD.

Overall, the inclusion of the former food product, Primo®, in the hens’ diet partially improved the nutritional quality of the eggs by (i) reducing the proportion of SFAs, generally considered detrimental for human health when consumed in high quantities (Maki et al., 2021), and (ii) increasing the proportion of C18:3n-3, considered to be beneficial (Simopolous, 2016). However, the increase in C18:3n-3, although significant, was rather limited, and did not affect the overall n-6/n-3 ratio.

In conclusion, the results of the present study support the inclusion of PBPI, namely the Primo® product, in laying hen diets as part of a more sustainable feeding strategy. The level of dietary inclusion, however, is critical in order to optimize productive efficiency. The lower inclusion level, corresponding to 20%, did not compromise total egg production, oviposition rate, or key egg quality traits such as EW, YW, AW, CP, and EE content. Furthermore, Primo® product inclusion was associated with improved egg quality from the nutritional standpoint as it reduced the total SFA content and increased C18:3n-3. Further trials are required to study the effect of the inclusion level in relation to diet composition, feeding method (*ad libitum vs* restricted), and hen age to support the valorization of PBPI as a feed ingredient in layer diet formulations and to promote the circular use of food resources.

## CRediT authorship contribution statement

**Niko Gioacchino Zeni:** Writing – original draft, Visualization, Validation, Methodology, Investigation, Data curation. **Vera Perricone:** Supervision, Investigation. **Cristina Tognoli:** Resources, Investigation. **Silvia Cerolini:** Writing – review & editing, Supervision. **Annalaura Lopez:** Writing – original draft, Visualization, Investigation. **Achille Schiavone:** Methodology, Conceptualization. **Stefano Paolo Marelli:** Validation, Formal analysis. **Andrea Giorgino:** Funding acquisition, Conceptualization. **Federico Fiamberti:** Conceptualization. **Paolo Rosso:** Validation, Formal analysis. **Luisa Zaniboni:** Project administration, Methodology, Funding acquisition, Formal analysis, Data curation, Conceptualization.

## Disclosures

The authors declare no conflicts of interest.
